# Sensor Arrays for Electrochemical Detection of PCR-Amplified Genes Extracted from Cells Suspended in Environmental Waters

**DOI:** 10.3390/s24227182

**Published:** 2024-11-08

**Authors:** Hiroshi Aoki, Mai Kawaguchi, Yukiko Kumakura, Hiroki Kamo, Kazuki Miura, Yuki Hiruta, Siro Simizu, Daniel Citterio

**Affiliations:** 1 Environmental Management Research Institute, National Institute of Advanced Industrial Science and Technology (AIST), 16-1 Onogawa, Tsukuba 305-8569, Ibaraki, Japan; kumakura-y@aist.go.jp; 2 Department of Applied Chemistry, Faculty of Science and Technology, Keio University, 3-14-1, Hiyoshi, Kohoku-ku, Yokohama 223-8522, Kanagawa, Japan; naomaik@keio.jp (M.K.); hiroki_kamo98@keio.jp (H.K.); k.miura@res.titech.ac.jp (K.M.); hiruta@applc.keio.ac.jp (Y.H.); simizu@applc.keio.ac.jp (S.S.)

**Keywords:** electrochemical DNA detection, sensor array, river water samples, environment

## Abstract

Ecological surveys of living things based on DNAs from environmental samples are attractive. However, despite simplicity of water sampling from the target environment, it is still necessary to transport the samples to the laboratory for DNA analysis based on skillful next-generation sequencers. To perform DNA-oriented surveys based on a simple protocol without any special training, we demonstrated, in this study, the detection of genes from cell-containing environmental waters using gene sensor arrays that require no DNA labeling and no external indicators. Cell-suspended PBS or river water were used as models of environmental waters containing living things, and DNA samples were prepared by PCR amplification. Ferrocene-terminated probes were synthesized and immobilized on an electrode array to develop a sensor array. The sensor array showed a large response to a target DNA complementary to the probe and no response to a mismatched DNA, indicating sequence-specific detection. For DNA samples prepared from the cells in PBS, they showed good responses similar to those for the target DNA. They also significantly detected DNA samples from the cells in river water at a general environmental concentration (38 cells mL^−1^) with 28-fold larger responses than those for 0 cells mL^−1^.

## 1. Introduction

With the UN’s sustainable development goals (SDGs) currently being promoted worldwide, numerous techniques are being developed to preserve the global environment by realizing recycling-oriented societies. Environmental preservation relies on investigative methods that detect living things in the environment [1]. Investigation of living things in the environment helps to obtain information that reveals the circumstances of the environment. Presently, as a way of evaluating environments, ecological surveys of living things based on DNA extracted from environmental samples are a growing focus of interest in the field of environmental studies on microorganisms [2,3,4], epidemiology [5,6], and environmental DNA (eDNA) [7,8,9]. This is a method of studying types of living things in the environment that targets the DNA from their cells or DNA excreted by them together with discharges and mucus. Conventional ecological surveys require living things to be captured and counted [10,11,12]. This method, however, by focusing on DNA, is considerably simpler in that all it requires is to sample the water of the target environment, since DNA analysis of water samples obtains information on the types of living things in the target environment [9,13,14,15,16]. This method helps to dramatically reduce the labor required for sampling compared to the conventional method [17,18].

For the comprehensive DNA analysis, next-generation sequencers (NGSs) used in comprehensive DNA analysis have been making remarkable advances and are in wide use [19]. However, these are analytical apparatuses that cannot be intuitively used by untrained personnel. Although collecting water samples, even from remote and rich natural environments, is a simple process, it is still laborious to transport the samples back to the laboratory for analysis. In addition, NGSs overperform DNA analysis if the target DNA is already known, especially, for example, when the living things to be investigated are specified in advance. For specific targets, portable real-time polymerase chain reaction (PCR) apparatuses have the potential to solve these problems. They are designed to detect DNA on-site and are seeing rapid improvements [20,21]. However, they have certain drawbacks, in that they require multiple reagents that can simultaneously target only a few sequences at the most. The amplification process currently needs a range of reagents, including fluorescence-labeled primers and probes, creating a tradeoff between the portability of the apparatus and the low number of target sequences per unit volume of the apparatus due to the spectroscopic detection principle. It should ideally be possible to perform DNA-oriented ecological surveys quickly, without the need for DNA labeling on-site, and using a simple protocol that requires no special training.

The authors have been studying electrochemical sensing techniques for detecting oligonucleotide sequences in sample solutions. Electrochemical oligonucleotide detection methods have the advantages of a simple design, compactness, and lower power requirements than other detection methods [22,23]. Moreover, to achieve faster and simpler oligonucleotide detection, we have developed techniques for detecting oligonucleotides without the need to label them [24]. They are based on an architecture in which the formation of a duplex between a target oligonucleotide and an oligonucleotide probe on the electrode’s surface triggers an electrochemical ON/OFF switch, revealing the presence of the target. Based on this architecture, we have promoted studies on architectures that detect changes in charge on the sensor surface [25], those using changes in the flexibility of the probe [26,27], and those based on the ON/OFF switching of an electroactive sensor surface controlled by an inhibitor-modified probe [28,29] when hybridization takes place. Architecture-based oligonucleotide sensor arrays have been applied to the sequence-specific detection of miRNA biomarkers for lung cancer and for mRNA biomarkers to detect exposure to estradiol, an endocrine disruptor, at the same time using a single sensor array chip [25,27]. Here, we thought that the use of the sensor array would help with the analysis of different species of DNA extracted from environmental water samples simultaneously on a single chip and that this would reveal the presence of target DNA in the samples.

In this study, aiming to achieve a comprehensive evaluation technique for DNA from living things in the environment, we demonstrated simple detection of genes from cell-containing environmental waters based on gene sensor arrays without the need for DNA labeling or any external indicators. In other words, as an important investigation prior to studies for eDNA detection based on real environmental samples, we investigated whether the sensor arrays can perform significant detection responses to DNA samples prepared from cell solutions with a general cell concentration in eDNA studies by using environmental waters and model cells with a defined cell concentration, utilizing the sensor array technique showing above-mentioned DNA detections. We used K562 human cells and prepared pseudo-real samples by suspending the cells in river water as a model of living things sampled from the environment. The samples were subjected to filtration to collect the cells, which were then disrupted to extract their genomic DNA. This extracted DNA was amplified using the polymerase chain reaction (PCR) to obtain DNA sample solutions. The 207 bp area from the β-actin gene was amplified using PCR. For use as oligonucleotide probes, we synthesized peptide nucleic acid (PNA) bearing ferrocene (Fc) at their distal terminal (Fc-PNA). The sensor arrays were prepared by immobilizing the probes on the electrodes of a photolithographically fabricated microelectrode array chip. The PNA sequence is specific to that of the β-actin gene. The prepared sensor array carrying the Fc-PNA showed a decrease in Fc oxidative signals after immersion in solutions containing complementary oligo DNA, leading to the detection of the DNA, whereas the array did not respond to mismatched oligo DNA. Moreover, for PCR-amplified DNA samples prepared from the cells suspended in PBS, the sensor arrays showed good responses similar to those for complementary oligo DNA. Finally, we successfully demonstrated that the developed array detected the PCR amplicons prepared from a pseudo-real sample of the cells suspended in river water based on our reported simple method for the processes of cell retrieval, genome extraction, and PCR amplification. To the best of our knowledge, this is the first demonstration of electrochemical gene sensor array-based detection of DNA extracted from cells suspended in environmental waters. We conclude from these results that the developed sensor array will greatly be expected to contribute to the simple detection of DNA or RNA in the field.

## 2. Materials and Methods

### 2.1. Reagents

**Scheme 1:**
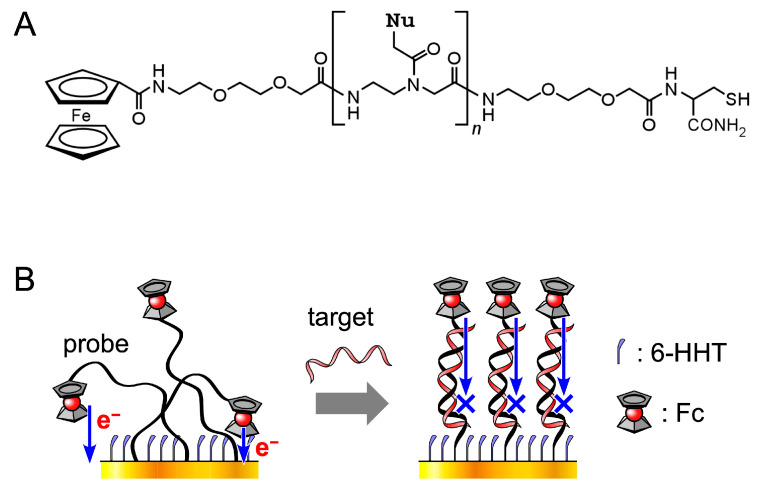
Molecular structure of a Fc-PNA probe used in this study (**A**). The Fc-PNA probe has a peptide nucleic acid (PNA) part for sequence-specific oligonucleotide recognition (hybridization), a terminal Fc moiety for signal generation, and a terminal cysteine moiety for immobilization on a gold electrode surface. Working principle of the Fc-PNA probe before and after hybridization (**B**). A terminal Fc reaches the electrode surface due to the flexible structure of the single-stranded probe before hybridization. After hybridization, the rigid structure of the double-stranded probe keeps the Fc away from the surface, showing the hybridization event as a decrease in the Fc redox current.

The oligo DNA was purchased from Thermo Fisher Scientific (Waltham, MT, USA) or Eurofins Genomic Japan (Tokyo, Japan). The synthesized oligonucleotide probes, purchased from Biologica (Nagoya, Japan), were designed as a conjugate of peptide nucleic acids (PNAs) and other moieties, with Fc, O, and Cys, respectively, denoting a ferrocene group, an ethylene glycol unit, and a cysteine group (Scheme 1A). Their sequences are summarized in Table S1. The sequences of Target DNA or Mismatch DNA are from that for the β-actin human genome DNA or for the genome DNA of a typical environmental microbe, Clostridium clostridioforme, respectively. 6-Hydroxy-1-hexanethiol (6-HHT) was from Dojindo (Kumamoto, Japan). Acetonitrile, glycerol, and 10× phosphate-buffered saline (10× PBS) were purchased from Fujifilm Wako Chemicals (Tokyo, Japan). 1× PBS was prepared from 10× PBS by 10-fold dilution. All aqueous solutions were prepared with deionized and charcoal-treated water (specified resistance > 18.2 MΩ cm), obtained using a Milli-Q reagent grade water system (Merck Millipore; Bedford, MA, USA).

### 2.2. Preparation of DNA Samples from Cells Suspended in PBS

We used samples from human leukemic K562 cells as a model cell suspended in PBS to investigate the performance of the prepared sensor arrays. The sample was prepared based on a 7.45 × 10^6^ cells mL^−1^ solution of the cells in PBS as described in our previously published paper [30]. The suspended cells were retrieved from 1 L of the sample using a syringe-based filtration system with a combination of a Kimwipe cellulose tissue (Nippon Paper Crecia; Tokyo, Japan) and a GF/F glass fiber filter (with a pore size of 0.7 µm and a thickness of 420 µm; GE Healthcare; Buckinghamshire, UK). The cell concentrations were confirmed using a microscope according to our previous report. DNA samples were prepared in our developed method, leading to completion within about 10 min, including on-filter cell lysis and DNA extraction, followed by DNA purification-free PCR amplification using extracted DNA on the filter as a template. The genome DNA of the cells was then directly extracted on filters by adding a 10 mL aliquot of 10 mM NaOH and 5 mL of 1 mM HCl to the filters. A 3 mm^2^ section cut out from the center of each filter was used as the template for the process of PCR amplification. We amplified a 207 bp portion of the exon part of the β-actin human genome DNA using the Ampdirect Plus enzyme set (Shimadzu Corporation; Kyoto, Japan) for direct PCR amplification from the template on the filter. A 50 µL aliquot of the sample solution was mixed in the PCR tube with 25 µL of 2× Ampdirect Plus, 0.25 µL of 5 U µL^−1^ BIOTAQTM HS DNA polymerase (Shimadzu), and 1 µL of 10 µM forward and reverse primer. The PCR process was performed by placing the PCR tube at 95 °C for 10 min, followed by 33 cycles at 94 °C for 30 s, 60 °C for 1 min, and 72 °C for 1 min. Finally, 7 min of heating was applied at 72 °C. The produced amplicons are depicted in Table S1 as Amplicon-1 and Amplicon-2. The amplification was confirmed by electrophoretic measurements on 2% agarose gel and fluorescence measurements on a Quantus Fluorometer (Madison, WI, USA). The fluorescence measurement with the Quantus Fluorometer found the concentration of DNA to be 6.1 ng µL^−1^. Assuming the molecular weights of Amplicon-1 and Amplicon-2 to be 63,441.9 and 64,359.6, respectively, the concentration of Amplicon-1 or Amplicon-2 was calculated to be 47.7 nM.

### 2.3. Preparation of DNA Samples from Cells Suspended in River Water as Pseudo-Real Samples

We prepared pseudo-real samples using river water by following the techniques described in our previously published paper, in which we developed a rapid pretreatment technique for the collection of DNA from K562 cells suspended in river water [30]. DNA collection and extraction from a river water sample can be completed within about 10 min. Briefly, pseudo-real samples were prepared from 0 cells mL^−1^ or 38 cells mL^−1^ suspended in river water (collected from the Yagami River in Kawasaki, Japan). Here, the actual DNA concentrations found for the salmonid fish Plecoglossus altivelis, commonly known as ayu, detected in the Saba River in Japan was used for reference and converted to a human K562 cell (estimated copy number 2) concentration of about 38 cells mL^−1^ [31]. The DNA samples were prepared in the same manner as mentioned in Section 2.2. Briefly, the suspended cells were retrieved from 1 L of the sample using a syringe-based filtration system. The cell concentrations were confirmed using a microscope according to our previous report. The genome extraction and PCR amplification processes were then performed. The amplification was confirmed by both electrophoretic and fluorescence measurements. The concentrations of the produced amplicons were 2.0 ng µL^−1^, corresponding to 15.6 nM.

### 2.4. Fabrication of Sensor Array Chips

**Figure 1:**
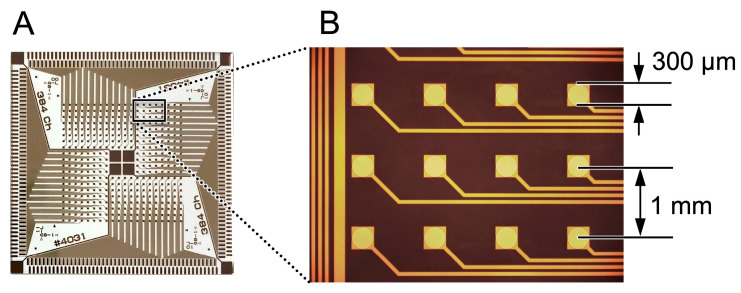
Photos of the prepared electrode array chip possessing 384 independent electrodes with a dimension of 36 mm square (**A**) and its magnified area having electrodes with a diameter of 300 µm and interval of 1 mm (**B**).

A 384 ch microelectrode array chip (384 channels, with an electrode diameter of 300 µm and an electrode interval of 1 mm, as shown in Figure 1) was fabricated in the manner previously reported [25,27]. Briefly, a glass substrate (36 mm × 36 mm; thickness 0.7 mm) was sputtered with titanium (thickness 1 nm) and gold (thickness 100 nm) thin films to form an Au/Ti film on the substrate. The Au/Ti-sputtered glass substrate was subjected to photolithography to pattern an electrode array. The chip has individual electrical terminals for each microelectrode for connection to an ALS 1030C electrochemical analyzer (Bioanalytical Systems (BAS); Tokyo, Japan). The chip was coated with an SU-8 3000 positive photoresist (thickness 10 µm; Kayaku Microchem; Tokyo, Japan) to act as electrical insulation, except for the surfaces of the microelectrodes and the electrical terminals.

A sensor array chip was prepared by modifying the electrode surfaces of the fabricated microelectrode array chip with Fc-PNA and 6-HHT as follows. The Fc-PNA was immobilized on the electrode surfaces of the chips by dropping a solution of 33 µM Fc-PNA in 1:1:1 1× PBS/acetonitrile/glycerol (*v*/*v*) onto the electrode surfaces. The chips were then stored at room temperature for 24 h. After washing with water, the aqueous solution of 1 mM 6-HHT was dropped onto the surfaces and the chips were kept at room temperature for 4 h. After washing with water again, the chips were subjected to electrochemical measurements. Aqueous solutions of 50 µM Target, Mismatch, or PCR amplicon in 1:1 1× PBS/glycerol (*v*/*v*) were then dropped onto the surfaces and the chips were incubated at room temperature for 12 h. The chips were then rinsed with water and subjected to electrochemical measurements.

In the modification and incubation processes, electrode areas were separated from each other by forming hydrophobic boundaries on the chip surfaces by stamping silicone rubber pieces (length 10 mm; thickness 3 mm; frame width 3 mm, as shown in Scheme S1 and Figure S1) onto the surfaces to soak different electrodes in solutions containing different DNA molecules.

### 2.5. Electrochemical Measurements

Electrochemical measurements were conducted using a three-electrode configuration that consisted of the prepared sensor as the working electrode, an Ag/AgCl reference electrode (internal solution: 3 M NaCl), and a Pt auxiliary electrode. They were carried out using an ALS 1030C electrochemical analyzer running its analytical software (BAS). The electrochemical measurements were performed using the same system as previously reported [25,27]. Briefly, the prepared sensor array chip was connected to the ALS 1030C with the electrical terminals formed on one edge of the chip using a chip adaptor with contact probes (Yokowo; Tokyo, Japan). The edge has 96 terminals, and each terminal is connected to a sensor on the chip. The electrochemical cell was constructed by placing a silicone rubber frame (size 28 mm × 28 mm; thickness 3 mm; frame width 3 mm) on the chip filled with 1 mL of the measurement solution, with the reference and auxiliary electrodes inserted into the solution perpendicularly and close to the chip (Figure S2).

Electrochemical measurements of sensor responses for the prepared sensor array chips were performed in 5 mM NaClO_4_ in 1× PBS at room temperature before and after incubation in solutions containing DNA. The sensor responses were evaluated by square wave voltammetry based on the decrease in oxidative peak current of the Fc moieties of the Fc-PNA probe. In the measurements, the potential was scanned from 0 to +0.65 V in potential steps of 2 mV, a potential amplitude of 25 mV, and a frequency of 50 Hz. Scheme 1B shows the working principle of the Fc-PNA probe before and after hybridization. The terminal Fc reaches the electrode surface due to the flexible structure of the single-stranded probe before hybridization. After hybridization, the rigid structure of the double-stranded probe keeps the Fc away from the surface, showing the hybridization event as a decrease in the Fc redox current. The sensor responses (*R*) to the DNA were expressed as the relative current change of (*i*_0_ − *i*)/*i*_0_ in the measured square wave voltammograms (SWVs), similar to our previous reports [25,27]. The relative current change was depicted in a pseudocolor scale ranging from green (low) to red (high). Here, the sensor responses over 1 or under 0 were rounded to 1 or 0, respectively. For each sample, the 12 sensor responses from 12 sensors were collected and the average ($\bar{R}$) and standard deviation (*s*) were calculated, as shown in Equations (1) and (2):

(1) $$\bar{R}=\frac{1}{12}\sum_{n}\frac{i_{0_{n}}-i_{n}}{i_{0_{n}}}=\frac{1}{12}\sum_{n}R_{n}$$

(2) $$s=\sqrt{\frac{1}{11}\sum_{n}{\left(R_{n}-\bar{R}\right)}^{2}}$$

The sensor responses are also used for the analysis based on a boxplot.

## 3. Results and Discussion

### 3.1. Separation of Electrode Areas by Stamping Silicone Rubber Pieces

The surface of the sensor array is hydrophilic, making it hard to keep solutions in their original positions for a long time and, therefore, preventing them from spreading. In the previous study, a hydrophobic adhesive film was used to separate the solutions to investigate multiple samples on a single sensor array, but the adhesion of the film to the surface tended to be weak during the incubation period, leading to intermixing of the solutions [25].

In this study, the electrode areas were separated by stamping silicone rubber pieces to form hydrophobic barriers that prevent the mixing of solutions during the incubation period (Figure S1A). In Scheme S1, hydrophobic barriers were made by placing silicone rubber pieces on the chip surface prior to dropping aqueous solutions on the electrode areas. Figure S1B shows photos of the sensor array after dropping the Fc-PNA or sample DNA solutions onto it. The Figures show that the solutions were kept separate during the processes and did not mix. This is because low molecular weight siloxane (poly-dimethylsiloxane with low molecular weight) seeped and transferred from the rubber pieces as a result of stamping them to form hydrophobic barriers [32,33]. Siloxane is chemically and electrochemically inert, and is transferred not onto the electrode surface but onto the SU-8 photoresist surface, due to the gap between these two surfaces (10 µm, the thickness of the SU-8). It should be pointed out here that there is no significant difference in sensor performance caused by the stamping of the silicone rubber pieces.

We describe below the detection of multiple samples based on this separation method.

### 3.2. Sensor Responses to Oligo DNA and PCR Amplicons on a Sensor Array

To evaluate the performance of the fabricated sensor arrays, we prepared sample solutions containing Target DNA (20-mer bases complementary to the Fc-PNA probe), Mismatch DNA (20-mer bases non-complementary to the probe), and PCR amplicons (207-mer bases complementary to the probe) (Table S1). The PCR amplicons were prepared by PCR amplification from the β-actin genome of the K562 cell suspended in PBS. For the incubation, aliquots of 5 µL of the solution were spread over the 12 sensor surfaces.

**Figure 2:**
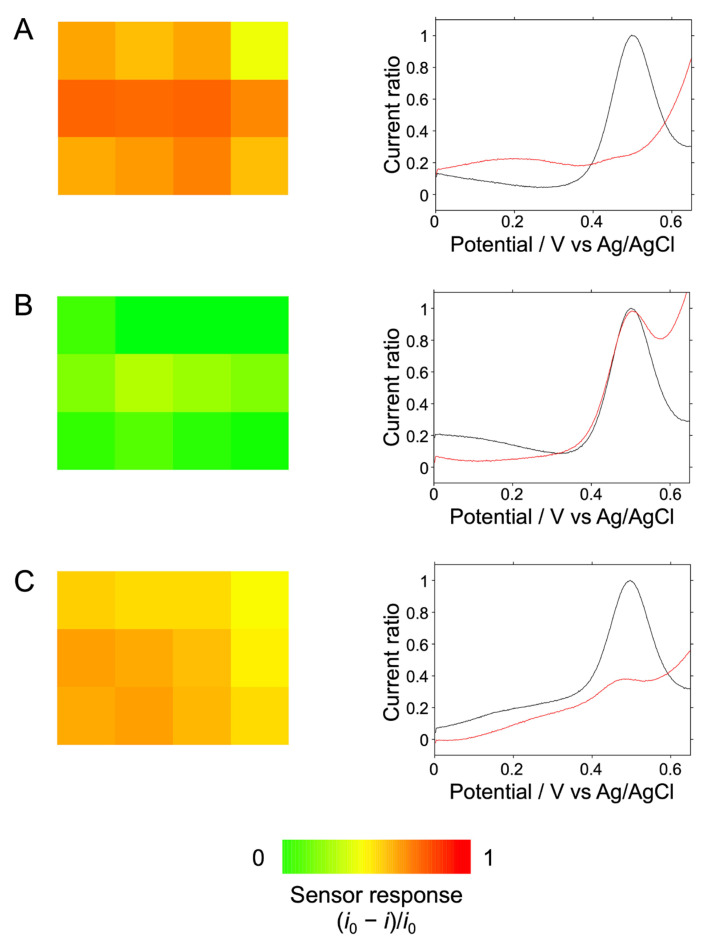
Sensor responses to the target DNA complementary to the probe (**A**), those to the mismatched DNA (**B**), or those to PCR amplified DNA prepared from oligonucleotides extracted from model cells to represent environmental DNA (**C**), expressing a level of decrease in Fc oxidative current in a pseudocolor scale (from green to red) (left). Typical SWVs before (black) and after (red) the incubations in the corresponding solutions are shown (right), where current values are normalized with the peak currents recorded before the incubation shown as current ratios.

The results are shown in Figure 2. Figure 2A shows the results of the sensor responses to Target DNA for a single sensor (right) and those for 12 sensors, expressed in a pseudocolor scale (left). In the result for the single sensor, a large oxidative peak was observed in the SWV after the immobilization of Fc-PNA, which dramatically decreased after incubation in the solution containing Target DNA. This indicates that the terminal Fc moiety of Fc-PNA can access the electrode surface, leading to electron transfer before incubation, and that the formation of a rigid duplex between the probe and the target inhibits Fc access and any subsequent electron transfer. The results for the 12 sensors, expressed in a pseudocolor scale, show that the sensor responses range from reddish orange to yellow. The average value of the sensor array responses was calculated as 0.629 ± 0.0966 (*n* = 12). This result demonstrates that the sensor array responded to Target DNA and agrees with the results of the previous reports [26,27].

The contrasting results for the sensor responses to Mismatch DNA for a single sensor (right) and those for the 12 sensors in a pseudocolor scale (left) are shown in Figure 2B. The oxidative peak current shown in SWVs for a single sensor remained almost unchanged before and after incubation in the solution containing Mismatch DNA, suggesting that the Fc-PNA probe did not form a duplex with Mismatch DNA. The results for the 12 sensors, expressed in a pseudocolor scale, show the senor responses to be mostly green. The average value of the sensor array responses was obtained as 0.0832 ± 0.101 (*n* = 12), revealing that the sensor arrays showed sequence-specific sensor responses to Target DNA and substantially no response to Mismatch DNA.

Finally, we investigated the sensor responses to the PCR-amplified 207 bp amplicon of the β-actin genome from the cells suspended in PBS. The PCR amplicons produced by the PCR amplification process are classified into two types of amplicons: Amplicon-1 and Amplicon-2 (sense and antisense sequences, respectively; shown in Table S1), where Amplicon-2 has a sequence complementary to the Fc-PNA probe. The concentration of the PCR amplicons (containing both Amplicon-1 and 2) was measured using the Quantus Fluorometer to be 6.1 ng µL^−1^ (95.4 nM), which shows the concentration of Amplicon-2 to be 47.7 nM. Figure 2C shows the result of this investigation. A single sensor showed a dramatic decrease in the peak current of the Fc oxidation before and after incubation, indicating duplex formation between the Fc-PNA probe and the amplicon. The sensor responses for the 12 sensors in a pseudocolor scale ranged from reddish orange to yellow. The average value of the sensor array responses was 0.541 ± 0.0634 (*n* = 12).

These results support those reported in our previous paper on sensor responses to complementary DNA using the sensors prepared from Fc-PNAs probes immobilized on gold disk electrodes [26]. The results of this study showed the sensor response to be 0.541 for the 47.7 nM DNA solution, whereas those of the previous study showed the relative peak current to be about 0.5, i.e., the sensor response was about 0.5 for the 10 nM (=10^−8^ M) DNA solution. The sensor arrays in this study therefore showed almost the same performance as the sensors previously prepared from gold disk electrodes, though the sequences and lengths used were different.

**Figure 3:**
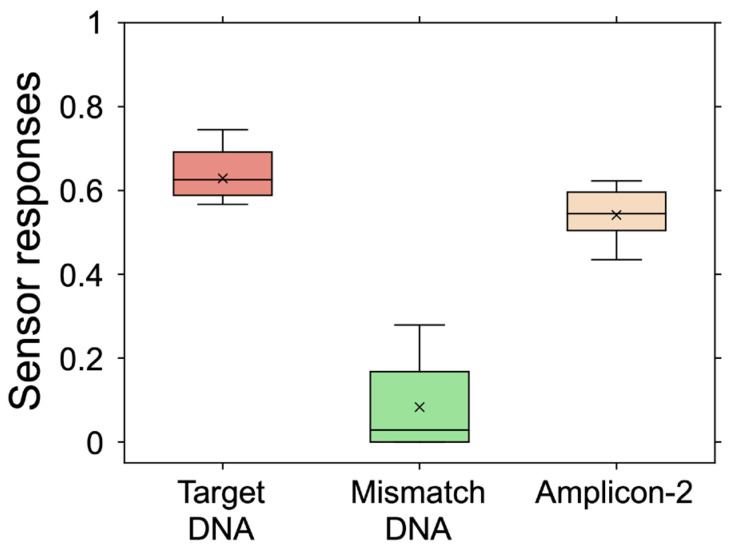
Boxplots of the sensor responses to Target DNA, Mismatch DNA, and Amplicon-2 (shown in Figure 2). The box edges are the 25th and 75th percentiles; the lower and upper error bars show the 10th and 90th percentiles, respectively; the horizontal lines inside the box show the median values; the cross marks denote mean values.

These results show that the prepared sensor array demonstrated sequence-specific sensor responses to complementary target sequences, as clearly represented in the boxplots in Figure 3. The PCR amplicon investigated in this study has a length of 207 bases, which is more than 10 times longer than the 20 bases of Target DNA. According to previous reports, regions other than the parts recognized by the Fc-PNA probe may interfere with the sensor surface during duplex formation between the amplicon and the probe immobilized on the surface [34,35]. Extremely long sequences would therefore appear to compromise sensor response. The part recognized by the Fc-PNA in Amplicon-2 is located from the 27th base to the 46th base at the 3′ terminal. The excess base length outside the part extending to the sensor surface is 26 bases. Most of the excess bases are designed to be located such that they extend into the bulk solution. Similar results were obtained in our previous study, in which DNA sensors with a probe PNA with 20 bases successfully detected PCR amplicons with 100 bases [25]. We conclude that, as designed, even a PCR product with 207 bases can form a duplex without significant physical interference on the sensor surface.

### 3.3. Cell-Containing River Water Samples as Pseudo-Real Environmental Samples

As a more demanding evaluation of the performance of the sensor arrays, a pseudo-real DNA sample was investigated in this section. We prepared the DNA sample from the river water in which 38 cells mL^−1^ of the model cells had been suspended, and conducted cell collection, DNA extraction, and PCR amplification. As mentioned in Section 2.3, the concentration was determined from the actual DNA concentration found for Plecoglossus altivelis (ayu), in the river [31]. The obtained PCR amplicons had both Amplicon-1 and Amplicon-2 sequences. The concentration of Amplicon-2 complementary to the probe was found to be 2.0 ng µL^−1^ (15.6 nM). As described in the previous section, aliquots of 5 µL of the solution were spread over the 12 sensor surfaces for incubation.

**Figure 4:**
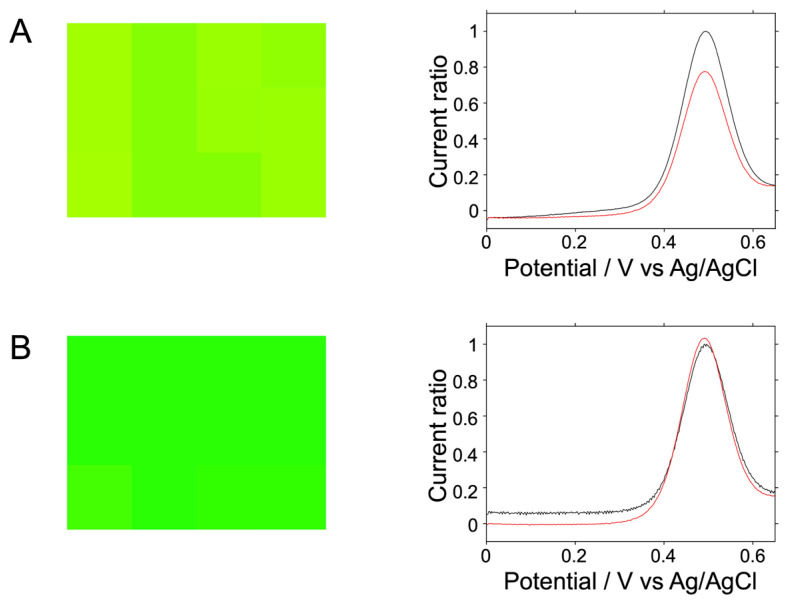
Sensor responses to the PCR amplicon amplified from samples prepared from the river water containing 38 cells mL^−1^ (**A**) or 0 cells mL^−1^ (**B**) with an incubation time of 12 h (**A**,**B**), expressing a level of decrease in Fc oxidative current in a pseudocolor scale (from green to red) (left). Typical SWVs before (black) and after (red) the incubations in the corresponding solutions are shown (right), where current values are normalized with the peak currents recorded before the incubation shown as current ratios.

The results are shown in Figure 4. The PCR amplicon prepared from the river water sample containing 38 cells mL^−1^ of the cells decreased the Fc oxidative peak currents for the Fc-PNA probe after incubation, and positive sensor array responses were observed (Figure 4A). The average value of the responses was 0.214 ± 0.0259 (*n* = 12). This result for the sample prepared from river water showed smaller responses than those for the sample from PBS (described in the preceding section). We concluded that this reflected the lower concentration of the cells and PCR amplicons in the sample from the river water (38 cells mL^−1^ and 15.6 nM, respectively) than those in the sample from PBS (7.45 × 10^6^ cells mL^−1^ and 47.7 nM, respectively). Our previous paper demonstrated that a sensor response of 0.2 showed a 0.1 nM level of DNA concentration and, conversely, a 10 nM DNA concentration should be expected as a sensor response of about 0.5 [26]. It can therefore be thought that the responses for the river water sample were smaller than those expected by referring to the previous paper. As one important factor, the smaller responses for the river water sample appear to also be due to the effects of some components in the sample added to promote the processes, including the direct DNA extraction/amplification to simplify the processes. There might be a trade-off relationship between simplicity of sample preparation and lower electrochemical response.

On the other hand, we used a sample prepared from river water containing 0 cells mL^−1^ sample, that is, river water only, subjected to the cell recovery, DNA extraction, and PCR amplification processes. The observed peak current was almost unchanged before and after incubation (Figure 4B). The average value of the sensor array responses was calculated to be 0.00767 ± 0.0150 (*n* = 12). Compared to this result, that for 38 cells mL^−1^ (0.214 ± 0.0259) was about 28-fold larger. Although the samples in the river water yielded smaller responses than those in the PBS, the sensor array could clearly distinguish between the samples of the cells from the river water. The obtained results indicate that the sensor array should be able to identify DNA in real environment samples.

**Figure 5:**
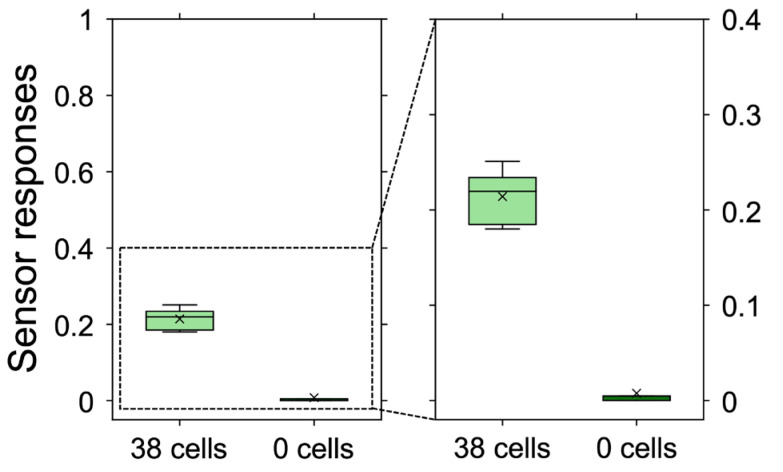
Boxplots of sensor responses to the PCR amplicon from samples prepared from river water containing 38 cells mL^−1^ or 0 cells mL^−1^ with an incubation time of 12 h (shown in Figure 4) in the same manner as that for Figure 3.

The prepared sensor array therefore demonstrated sequence-specific sensor responses to complementary target sequences, as clearly shown in Figure 5. The cell sample of 38 cells mL^−1^ could be detected and distinguished by comparing the results of the same process applied to the 0 cells mL^−1^ sample.

### 3.4. Limitations and Advantages of Sensor Arrays

Using the sensor array developed in this study, we aimed to develop a simple process for detecting the target sequence of the DNA samples prepared from cells suspended in PBS and in river water. The cell concentration of 38 cells mL^−1^ used in this study is a value calculated from the number of copies of DNA present in the actual environmental water. The comparison of the sensor responses to the DNA samples prepared from 0 cells mL^−1^ and 38 cells mL^−1^ cell samples yielded reproducible and significant results, indicating that the sensor array fabricated in this study is able to detect the concentrations of cells contained in real samples. To the best of our knowledge, this is the first demonstration of the electrochemical DNA sensor array-based detection of DNA extracted from cells suspended in environmental waters.

In this study, we used chips having the same microelectrode layouts as those used in our previous reports. The previous studies based on sensor arrays with the same layouts as those in this study demonstrated the detection of biomarker RNA sequences based on a probe PNA without Fc, requiring [Fe(CN)_6_]^4−^ as an external marker [25], and a methodology for electrode cleaning methods for long-term stored electrode array chips [27]. On the basis of these prior studies, we conducted the current study on the detection of cell-derived genomic DNA from environmental water via the Fc-PNA based-DNA detection method that does not require an external marker.

The DNA detection method used in this study is based on a ferrocene-bearing PNA probe as an electrochemical switch responsive to DNAs with specific sequences. This contributes to constructing an architecture for rapid and simple DNA detection. A limitation of this method is the requirement to focus on the target sequences and to prepare the probes prior to detection, while NGSs allow comprehensive DNA analysis. As mentioned in the Introduction, it is important to understand the advantages of the developed sensor arrays as focused DNA arrays and NGSs as comprehensive systems and to use them according to their purposes.

The present method based on sensor arrays achieved simplicity of DNA detection. In contrast, to bring out the best in the rapidity of the pre-treatment, reduction in the incubation time is the next challenge. While the incubation processes were performed for 12 h in this study for the detection of the DNA samples, the pre-treatment method in this study took only 10 min for the processes of cell retrieval and genome extraction from a river water sample [30]. Our previous reports demonstrated that an incubation time of 20 min is needed to perform hybridization between PNA and DNA [26], when the incubation temperature was kept at 65 °C. There is a possibility that temperature control during incubation could contribute to reducing the incubation time. According to these results, there is potential to reduce the time for the total process to analyze environmental samples to around 1 h.

When applying this method to real samples, it is expected that there are other issues to be addressed besides the above-mentioned temperature control. Matrix effects from complex real samples may affect the sensor response. For example, mine wastewater, seawater, and food-derived samples have issues such as the presence of heavy metal ions, various pH, and high salt concentrations, which were not observed in the river water used in this study. These issues may affect the hybridization efficiency and the stability of the monolayer on the sensor surface, and pretreatment appropriate for each sample will be necessary. As one of the examples, we have already demonstrated a pretreatment method for mine wastewater [36], decontaminating heavy metal ions and controlling the pH to around 7.

In this study, to drop multiple sample solutions onto the single sensor array chip, a hydrophobic barrier was formed by attaching a silicone rubber partitioning material to the chip surface. The hydrophobic barrier formed on the chip surface was found to effectively retain the droplets inside and outside the barrier during the incubation step and prevent the droplets from intermixing. In previous studies, a hydrophobic barrier was constructed by attaching a water-repellent film, but droplets inside and outside the barrier sometimes crossed over and mixed with the others during the incubation process [25]. This is because the adhesive layer between the surface of the sensor chip and the water-repellent film swells due to contact with the droplets, and as a result, the adhesive layer loses adhesiveness and the water-repellent film peels off from the surface. In this study, we addressed this problem by directly forming a hydrophobic barrier on the chip surface instead of using an adhesive water-repellent film. This process also greatly contributed to the simple detection of multiple DNAs on a single sensor array.

## 4. Conclusions

We have demonstrated simple detection of genes from cell-containing environmental waters based on gene sensor arrays without the need for DNA labeling or any external indicators, using our simple pre-treatment method for the processes of cell retrieval, genome extraction, and PCR amplification. We successfully achieved the ability to distinguish between DNA samples containing 38 cells mL^−1^ and those for 0 cells mL^−1^ of cells in river water with a significant sequence-specificity, showing a 28-fold larger response in the former cases. This is the first demonstration of gene sensor array-based detection of DNAs from cells suspended in environmental waters.

As a preliminary investigation, the sensor array developed in the present study is a promising technique. It can be applied to many other evaluation studies based on DNA or RNA biomarkers by using different sequences instead. We have a plan as the next step to move on to the study of eDNA sensor arrays for real environmental samples. Applications of the sensor arrays to eDNA detection will contribute to the identification of living things including fishes, microorganisms, and animals inhabiting target environments. The sensor arrays have great potential for on-site evaluation of environments including even remote or rich natural environments, because they are simple and free from the need for DNA labeling or any external indicators. For analysis of real environmental samples, sample preparation without the requirement for PCR amplification will contribute to a more rapid and simple assay. It is feasible to collect larger volumes of water to obtain more concentrated DNA samples.

The sensor arrays also have potential for application to biomedical fields, not only environmental fields. Moreover, they could also be possibly applied to the detection of not only DNAs but also RNAs. For instance, we have found several RNA biomarkers that are more responsive to stimuli from exposure to chemical substances than conventional ones in our study using mouse ES cells [37]. The use of probes specifically targeting these biomarkers allows these sensor arrays to be applied to the evaluation of chemical toxicity in the environment. We believe that our developed sensor array will greatly contribute to the on-site detection of DNA and RNA in the environmental field.

## Data Availability

The datasets generated during and/or analyzed during the current study are available from the corresponding authors upon reasonable request.

## Supplementary Materials

The following supporting information can be downloaded at https://www.mdpi.com/article/10.3390/s24227182/s1. Table S1: The sequences of the PCR primers, the PCR amplicon, and the probe PNA used in this study. In the Amplicon sequences, single underlines show the locations of forward and reverse primers and the double underline shows where it binds to the probe PNA. Scheme S1: Preparation of hydrophobic barriers by placing silicone rubber pieces on the chip surface on which to drop aqueous solutions on electrode areas. The silicone rubber pieces are placed on the chip surface (A) to create hydrophobic barriers on the surface (B). Aqueous solutions are dropped onto the electrode areas, where each drop is separately localized to each area (C). Figure S1: Photos of silicone rubber pieces that form hydrophobic barriers on the chip surface (A) and the prepared electrode areas for dropping the solutions of the probe or sample DNA (B) treated in the manner of Scheme S1. Figure S2: Experimental setup used in this study. The electrochemical cell is constructed by placing a silicone rubber frame on the chip filled with 1 mL of the measurement solution of 0.1 M NaClO4 in 1× PBS, with the Ag/AgCl reference and Pt auxiliary electrodes.
