# Supplementary material for: Sensor Arrays for Electrochemical Detection of PCR-Amplified Genes Extracted from Cells Suspended in Environmental Waters
Source: Sensors (Basel). 2024 Nov 8;24(22):7182. doi: 10.3390/s24227182 (PMC11598411; doi:10.3390/s24227182)

Supplementary Materials for

Sensor Arrays for Electrochemical Detection of PCR-Amplified Genes Extracted from Cells Suspended in Environmental Waters

Hiroshi Aoki [1,*], Mai Kawaguchi [2], Yukiko Kumakura [1], Hiroki Kamo [2], Kazuki Miura [2], Yuki Hiruta [2], Siro Simizu [2] and Daniel Citterio [2,*]

[1] Environmental Management Research Institute, National Institute of Advanced Industrial Science and Technology (AIST), 16-1 Onogawa, Tsukuba 305-8569, Ibaraki, Japan; kumakura-y@aist.go.jp

[2] Department of Applied Chemistry, Faculty of Science and Technology, Keio University, 3-14-1, Hiyoshi, Kohoku-ku, Yokohama 223-8522, Kanagawa, Japan; naomaik@keio.jp (M.K.); hiroki_kamo98@keio.jp (H.K.); k.miura@res.titech.ac.jp (K.M.); hiruta@applc.keio.ac.jp (Y.H.); simizu@applc.keio.ac.jp (S.S.)

* Correspondence: aoki-h@aist.go.jp (H.A.); citterio@applc.keio.ac.jp (D.C.)

List of captions

Table S1. The sequences of the PCR primers, the PCR amplicon, and the probe PNA used in this study. In the Amplicon sequences, single underlines show the locations of forward and reverse primers and the double underline shows where it binds to the probe PNA.

Scheme S1. Preparation of hydrophobic barriers by placing silicone rubber pieces on the chip surface on which to drop aqueous solutions on electrode areas. The silicone rubber pieces are placed on the chip surface (A) to create hydrophobic barriers on the surface (B). Aqueous solutions are dropped onto the electrode areas, where each drop is separately localized to each area (C).

Figure S1. Photos of silicone rubber pieces that form hydrophobic barriers on the chip surface (A) and the prepared electrode areas for dropping the solutions of the probe or sample DNA (B) treated in the manner of Scheme S1.

Figure S2. Experimental setup used in this study. The electrochemical cell is constructed by placing a silicone rubber frame on the chip filled with 1 mL of the measurement solution of 0.1 M $NaClO_4$ in 1× PBS, with the Ag/AgCl reference and Pt auxiliary electrodes.

Table S1. The sequences of the PCR primers, the PCR amplicon, and the probe PNA used in this study. In the Amplicon sequences, single underlines show the locations of forward and reverse primers and the double underline shows where it binds to the probe PNA.

Forward primer	5’ TCA TGT TTG AGA CCT TCA ACA C 3’
Reverse primer	5’ TCA TGA GGT AGT CAG TCA GG 3’
Amplicon-1	5’ TCA TGT TTG AGA CCT TCA ACA CCC CAG CCA TGT ACG TTG CTA TCC AGG CTG TGC TAT CCC TGT ACG CCT CTG GCC GTA CCA CTG GCA TCG TGA TGG ACT CCG GTG ACG GGG TCA CCC ACA CTG TGC CCA TCT ACG AGG GGT ATG CCC TCC CCC ATG CCA TCC TGC GTC TGG ACC TGG CTG GCC GGG ACC TGA CTG ACT ACC TCA TGA 3’
Amplicon-2	5’ TCA TGA GGT AGT CAG TCA GGT CCC GGC CAG CCA GGT CCA GAC GCA GGA TGG CAT GGG GGA GGG CAT ACC CCT CGT AGA TGG GCA CAG TGT GGG TGA CCC CGT CAC CGG AGT CCA TCA CGA TGC CAG TGG TAC GGC CAG AGG CGT ACA GGG ATA GCA CAG CCT GGA TAG CAA CGT ACA TGG CTG GGG TGT TGA AGG TCT CAA ACA TGA 3’
Target DNA	5’ TGG ATA GCA ACG TAC ATG GC 3’
Mismatch DNA	5’ TCC GCA GGC GGT TCC GCA AA 3’
Probe Fc-PNA	5’ Fc-O-GCC ATG TAC GTT GCT ATC CA-O-Cys 3’

Scheme S1. Preparation of hydrophobic barriers by placing silicone rubber pieces on the chip surface on which to drop aqueous solutions on electrode areas. The silicone rubber pieces are placed on the chip surface (A) to create hydrophobic barriers on the surface (B). Aqueous solutions are dropped onto the electrode areas, where each drop is separately localized to each area (C).

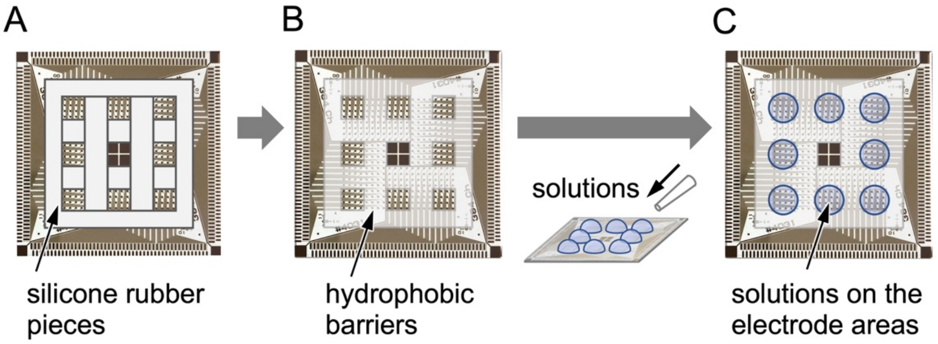

Figure S1. Photos of silicone rubber pieces that form hydrophobic barriers on the chip surface (A) and the prepared electrode areas for dropping the solutions of the probe or sample DNA (B) treated in the manner of Scheme S1.

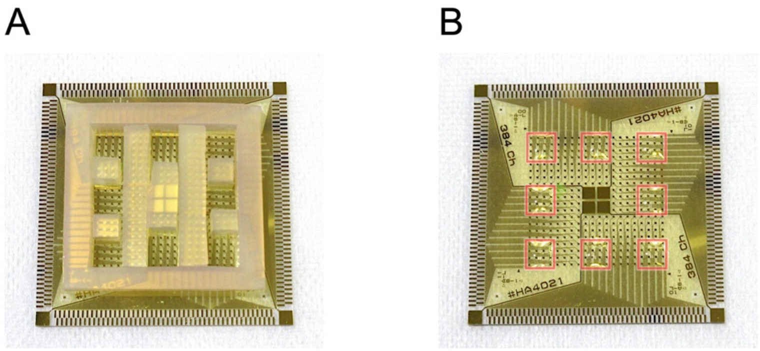

Figure S2. Experimental setup used in this study. The electrochemical cell is constructed by placing a silicone rubber frame on the chip filled with 1 mL of the measurement solution of 0.1 M $NaClO_4$ in 1× PBS, with the Ag/AgCl reference and Pt auxiliary electrodes.

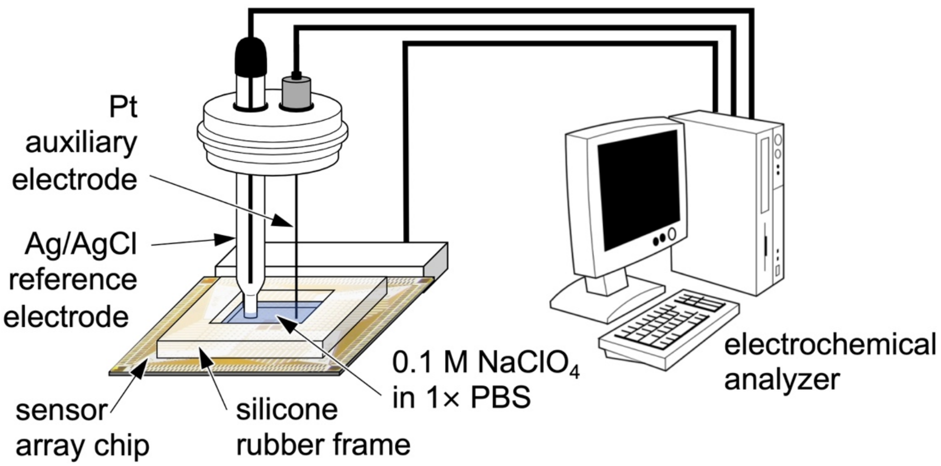

Supplement: Supplementary file 1 [file sensors-24-07182-s001.zip › sensors-3252353-supplementary.pdf]
